# Outcome for Patients with Triple-Negative Breast Cancer Is Not Dependent on Race/Ethnicity

**DOI:** 10.1155/2012/764570

**Published:** 2012-05-08

**Authors:** Quyen D. Chu, Amanda E. Henderson, Fred Ampil, Benjamin D. L. Li

**Affiliations:** ^1^Department of Surgery, Louisiana State University Health Sciences Center in Shreveport, Shreveport, LA 71103, USA; ^2^The Feist-Weiller Cancer Center, Louisiana State University Health Sciences Center in Shreveport, Shreveport, LA 71103, USA; ^3^Department of Radiology, Louisiana State University Health Sciences Center in Shreveport, Shreveport, LA 71103, USA

## Abstract

*Introduction*. Triple negative breast cancer (TNBC) is biologically aggressive and is associated with a worse prognosis. To understand the impact of race/ethnicity on outcome for patients with TNBC, confounding factors such as socioeconomic status (SES) need to be controlled. We examined the impact of race/ethnicity on a cohort of patients of low SES who have TNBC. *Methods*. 786 patients with Stage 0–III breast cancer were evaluated. Of these, 202 patients had TNBC (26%). Primary endpoints were cancer recurrence and death. ZIP code-based income tract and institutional financial data were used to assess SES. Data were analyzed using Kaplan-Meier survival analysis, log-rank tests, Cox Proportional hazard regression, chi square test, and *t*-tests. A *P* value ≤0.05 was considered statistically significant. *Results*. Of the 468 African-Americans (60%) in the database, 138 had TNBC; 64 of 318 Caucasians had TNBC. 80% of patients had an annual income of ≤$20,000. The 5-year overall survival was 77% for African-American women versus 72% for Caucasian women (*P* = 0.95). On multivariate analysis, race/ethnicity had an impact on disease-free survival (*P* = 0.027) but not on overall survival (*P* = 0.98). *Conclusion*. In a predominantly indigent population, race/ethnicity had no impact on overall survival for patients with triple negative breast cancer.

## 1. Introduction

Breast cancer is the most frequently diagnosed cancer in women and is associated with substantial morbidity and mortality. Breast cancer is a heterogeneous disease with different subtypes that are based upon the expression level of estrogen receptor (ER), progesterone receptor (PR), and HER-2/*neu* receptor (HER-2) [1]. Triple-negative breast cancers (TNBC), are breast tumors characterized by lack of expression of estrogen (ER), progesterone (PR), and HER2*neu* receptors and comprise 15% of all breast cancers. Most TNBC have a basal-like molecular phenotype by gene expression profiling [2, 3]. TNBC also shares clinical and pathologic features with hereditary BRCA1-related breast cancers including lack of ER/PR and HER2*neu*, presence of p53 mutation, basal gene expression patterns, and BRCA1 inactivation by either mutation or pathway dysfunction [4–6]. Most of these tumors are high grade or poorly differentiated tumors [7, 8]. TNBC has been shown to be associated with a poorer prognosis compared to receptor positive breast cancer subtype [9]. TNBC is not responsive to hormonal therapies such as Tamoxifen and aromatase inhibitors nor to inhibitors of HER-2 such as trastuzumab. Whether the poorer outcome with TNBC is the result of the loss of these therapeutic options or is the result of a more aggressive tumor biology or both is unknown.

African-American women have higher breast cancer death rates compared to Caucasian women, despite having a lower incidence of breast cancer [10]. Based on the 2001–2005 data from the Louisiana Tumor Registry, breast cancer mortality was 25.3% for Caucasian females and 40.9% for African-American females. The causes for this disparity in outcomes are not known. Some investigators believe that socioeconomic factors play a significant role in breast cancer disparities, while others speculate that it is the biologic differences that play a central role in outcome disparities. TNBC has been demonstrated to be more prevalent among young African-American females when compared to Caucasian women and is the basis for the argument in favor of biology as the culprit for breast cancer disparities. However, many of these studies do not adequately control for socioeconomic status (SES). Possibly related to SES is the observation that African American women are less likely to be diagnosed at an early stage when treatment can improve survival. Additional other potentially SES-related factors that may contribute to the survival difference include unequal access to medical care, health insurance status, treatment noncompliance, and socioeconomic status [11]. 

Louisiana State University Health Sciences Center in Shreveport (LSUHSC-S) is a public hospital and is unique in that the majority of the patients treated are uninsured or receive Medicaid and are of low socioeconomic status. We have previously demonstrated that at LSUHSC-S overall survival of breast cancer patients is similar between African-American and Caucasian women when controlled for SES. However, this study did not specifically evaluate whether such parity of outcome occurred in the subset of patients with TNBC. Our current study is aimed to address the following question: among patients with TNBC who are of similar socioeconomic status and given equal access to medical care, does the survival disparity between the racial/ethnic groups still exist?

## 2. Methods

A prospectively maintained breast cancer database was created in 1998. Details of this database have previously been reported [12]. Briefly, patients with stage 0 to 3 breast cancer who were treated before October 2008 were accrued and analyzed. We obtained approval to conduct the study from our Institutional Review Board. Of the 803 breast cancer patients in the database, we excluded 17 patients because of the patients belonging to other ethnicities (Hispanics or Asians) or having incomplete data. Of the remaining 786 patients, 468 patients were African-Americans and 318 patients were Caucasians. Triple-negative breast cancers (TNBCs) are defined as tumors that lack estrogen, progesterone, and HER-2 expressions. We identified 202 patients (25.7%) with TNBC. The majority of patients (~90%) were treated at FWCC/LSUHSC-S and the remaining patients were treated at a safety-net hospital, the EA Conway Hospital, a sister public hospital managed by LSUHSC-S. The American Joint Committee on Cancer (AJCC) 6th Edition was used to stage patients [13].

Two Society-of-Surgical-Oncology-(SSO) fellowship trained surgical oncologists performed the surgeries at FWCC/LSUHSC-S. Three general surgeons, each of whom had more than 10 years of surgical experience, performed surgeries at E. A. Conway Hospital. A weekly multidisciplinary tumor board conference was held to discuss all breast cancer cases performed for the previous week. Discussion of care of patients treated at E. A. Conway was conducted via telemedicine conferencing. Attendants of the weekly tumor board included a myriad of specialists (surgical oncologists, medical oncologists, radiation oncologists, radiologists, geneticists, residents, fellows, nurses, researchers, coordinators, and educators). 

All treatment and surveillance protocols were standardized in order to ensure study homogeneity. All patients were offered standard treatment protocols for adjuvant and neoadjuvant chemotherapy and radiation therapy. Antiestrogen therapy and herceptin were not used in this cohort. Definitive surgeries included either breast conservation therapy (BCT, lumpectomy with tumor-free margin, sentinel lymph node dissection and/or axillary lymph node dissection, and breast irradiation) or a mastectomy (±axillary lymph node dissection in select cases). After BCT, fractionated megavoltage external beam irradiation (encompassing the whole breast) to a total dose of 50 Gy/25 fractions was administered using tangential treatment portals; the supraclavicular area is irradiated (to the same total dose) when indicated (i.e., presence of disease to four or more axillary lymph nodes). Adjuvant systemic chemotherapy was offered and administered as indicated per current standard of care. 

Patient follow-up consisted of a history and physical examination every 3 months for 3 years, every 6 months for years 4 and 5, and annually thereafter. A chest X-ray, mammogram, complete blood count, and liver function tests were obtained annually. Additional radiologic and/or histologic evaluation was performed based on clinical indications. Clinical data were accrued and recorded prospectively and included age at diagnosis, comorbid conditions, stage of disease, treatment protocol, surveillance protocol compliance, cancer recurrence, and death. Compliance with treatment and surveillance protocols was over 90%.

Socioeconomic statuses were assigned to each patient based on two sources: the Internal Revenue Service 2001 ZIP-code-based income tract and the LSU Hospital Computer Service database. These sources did not differ between the two racial/ethnic groups and the data were not combined across methods. The Internal Revenue Service 2001 ZIP-code based income tract reports income as median annual income (MAI) per ZIP code stratified into quintiles based on ten thousand dollar increments. If the percentage of patients falls within 1% of either stratification group, the average of both groups was used to estimate the MAI. Because the 2001 tax year approximates the middle of dates of surgery for our patient population, the data from 2001 was chosen. All patients were assigned an MAI and stratified accordingly. 

Our hospital Computer Services database was used to link patients' financial code with their names, medical record numbers, initial dates of diagnosis, and ICD-9 diagnosis code 174.0–174.9. These financial codes were then used to stratify patients into the following subsets: commercial insurance, Medicare, Medicaid, or indigent/free care. Because this database only tracks patients for the past 7 years, only 57% of patients (115) were identified from this database. 

The impact of race/ethnicity on the outcome of patients with TNBC breast cancers was assessed by comparing outcomes between Caucasian and African-American women. Asian and Hispanic women comprised less than 5 patients in our large database and therefore were excluded from analysis. Clinical outcomes were then compared to five reports on outcome for patients with TNBC (Table 4) [1, 8, 14–16].

All statistical analyses were performed using MedCalc software (Microsoft, Inc.). The chi-square test was used to analyze categorical data, and the independent samples *t*-test was used to compare means. Disease-free survival (DFS) was calculated from the date of surgery to the date of first recurrence (local or distant) or date of last follow-up. Overall survival (OS) was calculated from the date of surgery to the date of death from any cause or date of last follow-up. 

The Kaplan-Meier survival method and the log-rank test were used to generate and compare survival curves. The Cox proportional hazard regression model was used to perform multivariate analyses. Risk ratios and 95% confidence intervals (CI) were calculated from the model. A *P *value ≤0.05 was considered statistically significant.

## 3. Results

Two-hundred and two patients with TNBC were identified. This represents approximately 26% (202/786) of all patients in our database.  Table 1 demonstrates patient, clinicopathologic, and socioeconomic characteristics of our cohort. There were 138 African-American women (68%) and 64 Caucasian women (32%) with TNBC representing 29% (138/468) of the African-American women and 20% (64/318) of the total number of Caucasian women in our database. The mean age at diagnosis was 54 years for African-American women and 60 years for Caucasian women (*P* = 0.38), and the mean follow-up time was 52.8 months. 

The median annual income by ZIPcode for the entire group of patients with TNBC was $16,577 (range, $15,367 to $36,788). The median annual income was $16,493 (range: $15,367 to $36,772) for African-American women and was $16,667 (range: $15,795 to $36,787) for Caucasian women. The differences between the median incomes were statistically significant (*P* < 0.001) although the magnitude of such differences does not appear to be clinically relevant. All patients resided within geographical areas with reported median annual incomes of $40,000 or less, and approximately 90% (181/202) were in areas with a reported median annual income of less than or equal to $30,000. The financial data at the time of diagnosis indicated no difference in the percent of patients with commercial insurance, Medicare, Medicaid, or free care (Table 1). 

Of all the clinicopathologic parameters examined, only tumor grade (*P* = 0.04), type of definitive operation (*P* = 0.01), and median annual income (*P* < 0.001) were significantly different between the two racial/ethnic groups. Mean age at diagnosis (*P* = 0.38), mean tumor size (*P* = 0.35), tumor size distribution (*P* = 0.25), nodal distribution (*P* = 0.50), stage distribution (*P* = 0.31), receipt of adjuvant therapy (*P* = 0.33), and financial class distribution (*P* = 0.69) were not significantly different between the two racial/ethnic groups (Table 1).

Overall, locoregional recurrences occurred in 13.8% (28 of 202 patients) of patients. The locoregional recurrence rate for African-American women was 20% (13/64) for Caucasian women (*P* = 0.08). Additionally, 41/202 (20.3%) of the entire TNBC cohort died by the time of last follow-up (December 2009) with a mortality rate of 20% (28/138) for African-American women and 20% (13/64) for Caucasian women (*P* = 0.85).

**Figure 1:**
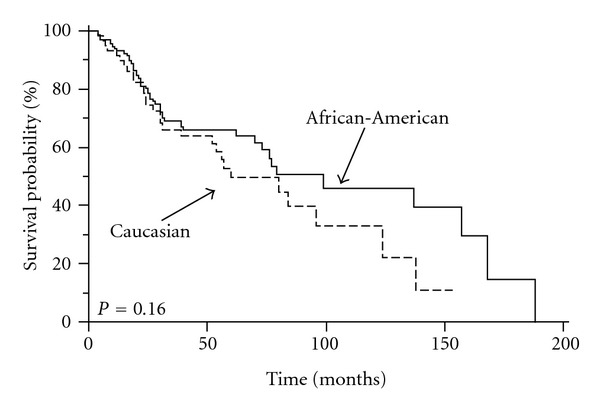
Effect of race/ethnicity on disease-free survival for 202 patients with triple-receptor negative breast cancer: shown is the DFS for 202 African-American and Caucasian patients with TNBC as described in section 2. The 5-year DFS was 66% for African-American women and 50% for Caucasian women (*P* = 0.16).

**Figure 2:**
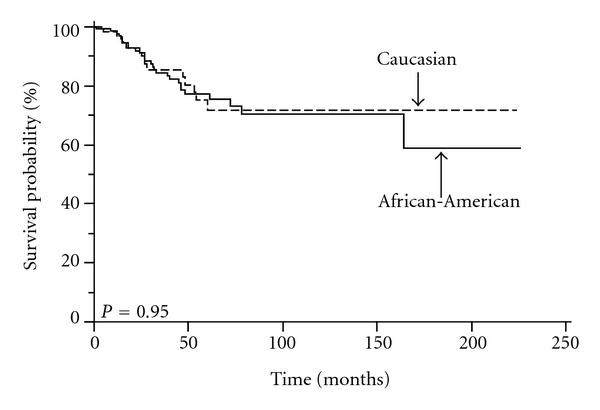
Effect of race/ethnicity on overall survival for 202 patients with triple-receptor negative breast cancer: shown is the OS for 202 African-American and Caucasian patients with TNBC as described in section 2. The 5-year OS was 77% for African-American women and 72% for Caucasian women (*P* = 0.95).

To discern the impact of race/ethnicity on the outcome for patients with TNBC, we evaluated OS and DFS between African-American and Caucasian women (Figures 1 and 2). In our previous studies, we demonstrated that neither OS nor DFS was significantly different between the two racial/ethnic groups, specifically in a large cohort of 786 patients with stage 0–3 breast cancers and a cohort of 375 patients with ER-negative tumors. Within the ER-negative tumors, we were able to identify a significant proportion of patients to have TNBC (54%). Therefore, this cohort was evaluated separately. 

Similar to our previous findings, in the subgroup of women with TNBC, we found no statistically significant difference in DFS or OS between the two racial/ethnic groups. The 5-year DFS was 66% for African-American women and 50% for Caucasian women; the median DFS was 99 months for African-American women and 60 months for Caucasian women (*P* = 0.16) (Figure 1). The 5-year OS was 77% for African-American women and 72% for Caucasian women; the median OS was 138 months for African-American women and 64 months for Caucasian women (*P* = 0.95) (Figure 2).

The Cox proportional hazard model was used to compare race/ethnicity, age at diagnosis, tumor grade, median income, T-stage, and N-stage for risk of cancer recurrence and overall survival (Tables 2 and 3). Note that although race/ethnicity was an independent predictor for DFS (*P* = 0.027), it was not an independent predictor for OS (*P* = 0.98). Clinical independent predictors for DFS were T-stage (*P* = 0.001) and N-stage (*P* = 0.05). Only N-stage (*P* = 0.01) was an independent predictor for OS. 

Suboptimal results run the risk of masking any potential significant differences in outcomes between African-American women and Caucasian women. Therefore, we compared our outcomes for women with TNBC with outcomes reported in the literature [1, 8, 14–16]. In selected published series, the 5-year OS rate ranges from 59.6% to 80% and the 5-year DFS rate ranges from 30.8% to 72%. Our figures compare favorably with these historic figures (Table 4).

## 4. Discussion

African-American women have a lower incidence of breast cancer but a higher breast cancer mortality rate when compared to Caucasian women [17–19]. Such disparity has been the focus of recent debates. Confounding variables make it difficult to establish the exact nature of such disparity. While some investigators attribute it to differences in income and social status, which affect access to and receipt of treatment, others accredit it to racial/ethnic differences in tumor biology and responsiveness to treatment [10–12, 14, 17, 18, 20–23]. Race/ethnicity as an independent predictor of survival in breast cancer has been reported in several studies, although most do not adequately control for socioeconomic status (SES) and/or tumor subtype (i.e., TNBC) [16]. 

In our initial study of 786 patients with operable breast cancer (stage 0–III), we demonstrated that race/ethnicity had no impact on outcome when equal access was rendered, regardless of patients' financial statuses. In that study, outcomes at LSUHSC-S rivaled those reported by the National Cancer Data Base (NCDB). These results were achieved in a population that has historically been associated with poorer outcomes; over two-thirds of our patients were classified as having either Medicaid or free care and the median annual income for both groups was less than $17,000 [12]. 

A potential confounder of the above study was an imbalance of the different breast cancer subtypes between African-American and Caucasian women. We noted that African-American women had a significantly higher proportion of ER-negative tumors than Caucasian women. To address this, we separately evaluated outcomes for 375 patients with ER-negative breast cancers to determine whether there was disparity between the two racial/ethnic groups [11]. However, similar to the results of our initial study, we found that there were no significant differences in breast cancer mortality rates between African-American and Caucasian women who had ER-negative tumors [11]. Again, these results were achieved in a relatively homogenous cohort of patients with low SES. 

One of the limitations of our ER-negative study was that it did not delineate the proportion of patients who had TNBC. TNBC is used by clinicians in reference to the basal-like subtype of breast cancer although only 85% of TNBCs are basal-like. Numerous studies have shown that TNBC is associated with a decreased overall survival when compared to receptor-positive tumors and that TNBC is more prevalent among African American women [24]. This fact has popularly been thought of as being one of the major contributors of disparity in outcomes between African-American women and Caucasian women [24]. However, our results demonstrated that even within the TNBC cohorts race/ethnicity had no impact on outcome. These results were obtained despite African-American women having had a significantly higher tumor grade than Caucasian women (grade 3 = 68% versus 51%; *P* = 0.04) and that TNBC was more predominant among African-American women than Caucasian women.

The principle that race/ethnicity should have no impact on outcome for patients with TNBC was further reinforced by a study by Dawood et al. [8]. In this study of nearly 500 patients who were treated with primary systemic chemotherapy followed by definitive surgery, neither pathologic complete response rates (pCR) nor survival outcomes differ between the two racial/ethnic groups [8]. 

The five-year overall survival rate for all breast cancer subtypes is approximately 89% and this rate drops precipitously for patients with TNBC (77% to 80%) [1, 16]. Our data seemed to support these results. What is unique about our cohort is that we were able to control for socioeconomic status and receipt of systemic therapy, thus eliminating any potential socioeconomic biases. 

Based on our previous and current data and findings, we can conclude that disparity in survival between African-American females and Caucasian females can be mitigated when all patients are provided with the same standard of care breast cancer treatment. This paradigm seems to be applicable for wide variety of breast cancer, including those with TNBC. In addition, our data do not support the idea of biological differences in tumor subtypes between Caucasian and African-Americans. The higher proportion of younger African-Americans developing TNBCs compared with Caucasians may still contribute to the overall worse outcomes, even though the responses to treatment are similar.

## Figures and Tables

**Table 1:** Distribution of patient, clinicopathologic, and socioeconomic characteristics of 202 Patients with triple negative breast cancer.

	African-American	Caucasian	*P* value
	(*N* = 138)	(*N* = 64)	
	68%	32%	
Characteristics			

Mean age years (range)	54 (28–33)	60 (36–87)	0.38

Mean tumor size (cm)	3.39	3.16	0.35

Tumor size distribution			
T1 (28%)	33 (24%)	24 (38%)	
T2 (53%)	76 (55%)	30 (47%)	0.25
T3 (13%)	19 (14%)	7 (11%)	
T4 (6%)	10 (7%)	3 (4%)	

Nodal distribution			
N0 (55%)	71 (51%)	40 (62%)	
N1 (25%)	37 (27%)	14 (22%)	0.5
N2 (15%)	23 (17%)	7 (11%)	
N3 (5%)	7 (5%)	3 (5%)	

Stage distribution			
Stage 1 (21%)	25 (18%)	17 (26%)	
Stage 2 (52%)	73 (53%)	33 (52%)	0.31
Stage 3 (27%)	40 (29%)	14 (22%)	

Tumor grade			
I/II (38%)	40/125 (32%)	29/59 (49%)	0.04
III (62%)	85/125 (68%)	30/59 (51%)	

Definitive surgery			
Breast-conserving Rx (31%)	51 (37%)	12 (19%)	0.01
Mastectomy (69%)	87 (63%)	52 (81%)	

Systemic treatment			
Adriamycin alone (19%)	28 (20%)	11 (17%)	
Adriamycin + Taxane (41%)	60 (44%)	22 (34%)	
Taxane alone (3%)	3 (2%)	3 (5%)	0.33
Hormone therapy alone (3%)	2 (1%)	3 (5%)	
Hormone therapy + chemotherapy (16%)	19 (14%)	14 (22%)	
Others (18%)	26 (19%)	11 (17%)	

Median annual income	$16,493	$16,667	
Mean (range) annual income	$17,873	$21,081	<0.001
	($15,367–$36,772)	($15,795–$36,787)	

Financial class			

Commercial (11%)	8/80 (10%)	5/35 (14%)	

Medicare (10%)	7/80 (9%)	4/35 (11%)	0.69

Medicaid (6%)	6/80 (7%)	1/35 (3%)	

Free care (73%)	59/80 (74%)	25/35 (72%)	

**Table 2:** Effect of race/ethnicity on cancer recurrence for patients with triple-receptor-negative breast cancer (Cox proportional hazard model).

	Relative Risk	95% CI	*P* value
Race/ethnicity	1.84	1.07 to 3.14	0.027
Age at diagnosis	1.00	0.98 to 1.03	0.80
Grade	1.20	0.71 to 2.03	0.49
Income level	0.87	0.54 to 1.40	0.58
T-stage	1.68	1.22 to 2.30	0.001
N-stage	1.29	1.0 to 1.68	0.05

**Table 3:** Effect of race/ethnicity on overall survival for patients with triple-receptor negative breast cancer (Cox proportional hazard model).

	Relative risk	95% CI	*P* value
Race/ethnicity	1.00	0.48 to 2.06	0.98
Age at diagnosis	1.01	0.98 to 1.05	0.41
Grade	1.89	0.89 to 3.97	0.09
Income level	1.15	0.63 to 2.09	0.65
T-stage	1.43	0.95 to 2.17	0.09
N-Stage	1.53	1.09 to 2.16	0.01

**Table 4:** Comparison of clinical outcomes for patients with triple-receptor negative breast cancer.

	Overall survival (%)	Disease free survival (%)
Chu (FWCC)	75	60; 66(AA), 50 (C)
Haffty	80	72
Bauer	77	—
Kyndi	50 (high-risk cohort)	—
Lund	59.6	30.8
Dawood	71 (3-yr OS)	68 (AA), 62 (C)

FWCC: Feist-Weiller Cancer Center, AA: African-american, and C: Caucasian.

## References

[1] Haffty BG, Yang Q, Reiss M (2006). Locoregional relapse and distant metastasis in conservatively managed triple negative early-stage breast cancer. *Journal of Clinical Oncology*.

[2] Perou CM, Sørile T, Eisen MB (2000). Molecular portraits of human breast tumours. *Nature*.

[3] Kreike B, van Kouwenhove M, Horlings H (2007). Gene expression profiling and histopathological characterization of triple-negative/basal-like breast carcinomas. *Breast Cancer Research*.

[4] Turner N, Tutt A, Ashworth A (2004). Hallmarks of ’BRCAness’ in sporadic cancers. *Nature Reviews Cancer*.

[5] Turner NC, Reis-Filho JS (2006). Basal-like breast cancer and the BRCA1 phenotype. *Oncogene*.

[6] Turner NC, Reis-Filho JS, Russell AM (2007). BRCA1 dysfunction in sporadic basal-like breast cancer. *Oncogene*.

[7] Kwan ML, Kushi LH, Weltzien E (2009). Epidemiology of breast cancer subtypes in two prospective cohort studies of breast cancer survivors. *Breast Cancer Research*.

[8] Dawood S, Broglio K, Kau SW (2009). Triple receptor—negative breast cancer: the effect of race on response to primary systemic treatment and survival outcomes. *Journal of Clinical Oncology*.

[9] Mersin H, Yildirim E, Berberoglu U, Gülben K (2008). The prognostic importance of triple negative breast carcinoma. *Breast*.

[11] Chu QD, Burton G, Glass J, Smith MH, Li BDL (2010). Impact of race/ethnicity on outcome for ER-negative breast cancers: experience of an academic center with a charity hospital. *Journal of the American College of Surgeons*.

[12] Chu QD, Smith MH, Williams M (2009). Race/ethnicity has no effect on outcome for breast cancer patients treated at an academic center with a public hospital. *Cancer Epidemiology Biomarkers and Prevention*.

[13] American Joint Committee on Cancer (2002). *AJCC Cancer Staging Manual*.

[14] Lund MJB, Butler EN, Bumpers HL (2008). High prevalence of triple-negative tumors in an urban cancer center. *Cancer*.

[15] Kyndi M, Sørensen FB, Knudsen H, Overgaard M, Nielsen HM, Overgaard J (2008). Estrogen receptor, progesterone receptor, HER-2, and response to postmastectomy radiotherapy in high-risk breast cancer: the Danish Breast Cancer Cooperative Group. *Journal of Clinical Oncology*.

[16] Bauer KR, Brown M, Cress RD, Parise CA, Caggiano V (2007). Descriptive analysis of estrogen receptor (ER)-negative, progesterone receptor (PR)-negative, and HER2-negative invasive breast cancer, the so-called triple-negative phenotype: a population-based study from the California Cancer Registry. *Cancer*.

[17] Jemal A, Siegel R, Ward E (2008). Cancer statistics, 2008. *CA: A Cancer Journal for Clinicians*.

[18] Newman LA, Griffith KA, Jatoi I, Simon MS, Crowe JP, Colditz GA (2006). Meta-analysis of survival in African American and white American patients with breast cancer: ethnicity compared with socioeconomic status. *Journal of Clinical Oncology*.

[19] Woodward WA, Huang EH, McNeese MD (2006). African-American race is associated with a poorer overall survival rate for breast cancer patients treated with mastectomy and doxorubicin-based chemotherapy. *Cancer*.

[20] Chlebowski RT, Chen Z, Anderson GL (2005). Ethnicity and breast cancer: factors influencing differences in incidence and outcome. *Journal of the National Cancer Institute*.

[21] Albain KS, Unger JM, Crowley JJ, Coltman CA, Hershman DL (2009). Racial disparities in cancer survival among randomized clinical trials patients of the southwest oncology group. *Journal of the National Cancer Institute*.

[22] Brawley OW, Berger MZ (2008). Cancer and disparities in health: perspectives on health statistics and research questions. *Cancer*.

[23] Huo D, Ikpatt F, Khramtsov A (2009). Population differences in breast cancer: survey in indigenous african women reveals over-representation of triple-negative breast cancer. *Journal of Clinical Oncology*.

[24] Stead LA, Lash TL, Sobieraj JE (2009). Triple-negative breast cancers are increased in black women regardless of age or body mass index. *Breast Cancer Research*.

